# Study on Microstructural Characterization, Mechanical Properties and Residual Stress of GTAW Dissimilar Joints of P91 and P22 Steels

**DOI:** 10.3390/ma14216591

**Published:** 2021-11-02

**Authors:** Anupam Sauraw, Atul Kumar Sharma, Dariusz Fydrych, Sachin Sirohi, Ankur Gupta, Aleksandra Świerczyńska, Chandan Pandey, Grzegorz Rogalski

**Affiliations:** 1 Department of Mechanical Engineering, Indian Institute of Technology Jodhpur N.H. 62, Nagaur Road, Karwar, Jodhpur 342037, India; sauraw.1@iitj.ac.in (A.S.); atulksharma@iitj.ac.in (A.K.S.); ankurgupta@iitj.ac.in (A.G.); jscpandey@iitj.ac.in (C.P.); 2 Institute of Manufacturing and Materials Technology, Faculty of Mechanical Engineering and Ship Technology, Gdańsk University of Technology, Gabriela Narutowicza Street 11/12, 80-233 Gdańsk, Poland; dariusz.fydrych@pg.edu.pl (D.F.); grzegorz.rogalski@pg.edu.pl (G.R.); 3 Mechanical Department, SRM Institute of Science and Technology, Delhi NCR Campus, Modinagar 201204, India

**Keywords:** P91 steel, P22 steel, ERNiCrMo-3, GTAW welding, dissimilar welded joint, microstructure, mechanical properties, stress

## Abstract

This article deals with the dissimilar joining of two different grade Cr-Mo steel (2.25Cr-1Mo: P22 and modified 9Cr-1Mo: P91) for power plant application. The dissimilar butt-welded joint was produced for conventional V groove design by using the gas tungsten arc welding (GTAW) process with the application of an ERNiCrMo-3 Ni-based super alloy filler. A microstructure characterization was performed to measure the inhomogeneity in the microstructure and element diffusion across the interface in a welded joint. The experiments were also performed to evaluate the mechanical properties of the dissimilar welded joint in as-welded (AW) and post-weld heat treatment (PWHT) conditions. An acceptable level of the mechanical properties was obtained for the AW joint. After PWHT, a significant level of the element diffusion across the interface of the weld metal and P22 steel was observed, resulting in heterogeneity in microstructure near the interface, which was also supported by the hardness variation. Inhomogeneity in mechanical properties (impact strength and hardness) was measured across the weldments for the AW joint and was reduced after the PWHT. The tensile test results indicate an acceptable level of tensile properties for the welded joint in both AW and PWHT conditions and failure was noticed in the weak region of the P22 steel instead of the weld metal.

## 1. Introduction

Super-critical power plants operate in the temperature range of 550–620 °C and consist of several dissimilar welded joints in order to minimize overall operating cost [1,2]. Power plant components such as boiler tubes, which operate in a temperature range of 500–550 °C, are generally made of low Cr steel ferritic/pearlitic steel, i.e., P22 steel [3]. The re-heater and super-heater tubes or steam-header section, having operating tempering in the range of 550–620 °C, are generally made of high Cr steel ferritic/martensitic like P91, P92 or P911 steel [4]. P22 steel exhibits a ferritic/pearlitic or ferritic/bainitic microstructure depending on it’s chemical composition and processing routes and offers poor creep strength and steam oxidation resistance as compared with P91 steel. P91 steel, having a ferritic/martensitic microstructure, derives its strength from its tempered martensitic microstructure and carbide and carbonitrides precipitates [5,6]. P91 steel offers good creep strength, mechanical properties and microstructure stability which requires higher steam operating temperature. P91 steel is also well known for its good thermal conductivity and lower thermal expansion coefficient, which helps to minimize the structural integrity problem during welding. P91 steel also offers excellent resistance to corrosion cracking and oxidation up to temperatures of 610 °C [7]. The poor oxidation resistance of P91 steel beyond 610 °C limits its application for power plant components operating at a temperature more than 610 °C [8].

Therefore, this requirement requires dissimilar welding of multi-material with superior integrity. Though dissimilar welding of P91 and P22 steel has been practiced for high-temperature application in power plants, failures have been reported from the heat-affected zone (HAZ) of P22 steel or from the interface region of the weld metal and P22 steel HAZ [9,10]. The major reason cited for these failures is a mismatch in the thermal expansion coefficient (CTE) of these two steels and metallurgical unsuitability such as diffusion of elements, inhomogeneity in microstructure along weldments, and the uncertain chemistry of the weld metal due to direct intermixing of the two materials during the joining of the P91 and P22 steel [11,12]. The variation in CTE (P22 steel: 14 × 10^−6^/K; P91 steel: 13.2 × 10^−6^/K) causes the evolution of thermal stresses along the welded joint, which might be the cause of the reduction in stress rupture strength. Diffusion of the carbon from P22 steel to weld metal during the PWHT or in-service condition also creates the heterogeneity in the microstructure along the interface and reduces the creep rupture life of the welded components [13,14,15]. Earlier research recommends the use of an inert-metallic layer or Ni-based superalloy filler to minimize the above metallurgical problem [16,17,18].

A multi-pass dissimilar joint of P91 and P22 steel is mainly produced by using a matching P91 filler due to its higher strength over a P22 filler. The use of a P91 filler mainly results in the formation of an untempered lath martensitic microstructure in the weld fusion zone, which shows poor impact toughness. Autogenous TIG and activated TIG joints of P22/P91 steel also produce poor impact toughness of the weld metal due to the formation of brittle untempered martensite [11,19]. The PWHT of the dissimilar welded joint of P91 and P22 steel was recommended by many of the researchers to increase the impact strength by tempering the martensite [11,20,21,22]. However, PWHT of the welded joint leads to the diffusion of elements across the interface of low Cr P22 steel and high Cr weld metal. The diffusion of the elements across the interface is a time and temperature control phenomenon and results in the formation of a soft zone (SZ) and hard zone (HD) near the interface [12,14,23]. The SZ and HZ formed near the interface are mainly measured in microns, and heterogeneity in microstructure in such a small region again provides crack nucleation sites during the service condition.

The precipitation of carbides in a hard zone of the dissimilar welded joint of P22 and P91 steel as a consequence of PWHT was confirmed by Sudha et al. [15]. A detailed examination of the mechanism of SZ and HZ formation along with precipitation sequence and chemistry of the carbides in a hard zone was studied. To calculate the composition of the carbides, an indirect approach was developed which was based on the type, amount and chemistry of the carbide precipitates. Sudha et al. [14] further continued their study on the growth mechanism of SZ and HZ for varying tempering times and reported a considerable effect of PWHT duration on the width and hardness of the SZ and HZ. An increase in width and decrease in hardness of the HZ and SZ, corresponding with an increase in PWHT duration, was observed. The primary factor responsible for the formation of SZ and HZ was a diffusion of the C from P22 to P91 steel driven by the gradient in C activity. Albert et al. [21] also performed a study on C migration along the interface of 9Cr-lMo steel weld metal and 2.25Cr-1Mo steel for varying PWHT times and temperatures. Like a previous study by Sudha et al. [14], Albert et al. [21] also found a significant effect of PWHT time on the width of the SZ and HD. However, an effect of the PWHT temperature on SZ and HZ was also studied by Albert et al. [21], who reported a considerable increase in size with an increase in PWHT temperature. To mitigate the problem of C diffusion, the selection of the filler consumable should be based on carbon activity data as suggested by Albert et al. [21]. The effect of the PWHT temperature on the carbon diffusion distance has also been examined by Sultan et al. [12]. The test was conducted for varying PWHT durations and reported a significant increase in the size of the carbon migration zones as PWHT duration increased from room temperature to 770 °C. However, a growth rate for the SZ was measured as higher than that of the HZ. Pandey et al. [20] studied the mechanical properties of the welded joint of P22/P91 steel in as-welded (AW) and PWHT duration and reported heterogeneity in mechanical properties, i.e., hardness and toughness. The high-temperature tensile behavior of the dissimilar welded joint of T23 and T91 steel showed that the grain boundary slides and that the cluster formation of M_23_C_6_ precipitates as it converges around prior austenite grain boundaries (PAGBs), resulting in cavity formation and ultimately final cleavage mode of fracture [22]. The creep behavior of the dissimilar Shielded Metal Arc Welded (SMAW) joint of P22/P91 steel was performed at a temperature of 550 °C in a stress range of 100–260 MPa for both AW and PWHT conditions [24]. The creep strength order was as follows: P91 steel weld metal ˃ P91 steel ˃ P22 steel ˃ dissimilar weld metal of P91 and P22 steel. The poor creep strength of the dissimilar weld metal as compared with both the base metals (P22 and P91 steel) was attributed to higher creep strain concentration in the region of SZ and the inter critical heat affected zone (ICHAZ) of P22 steel. The size of the carbon migration zones (SZ and HZ) and the hardness difference of HZ and SZ was observed to increase with creep exposure time.

The literature shows that the SZ and HZ formed during the PWHT showed an adverse effect on the long-term creep performance of the welded joint and the size and hardness of these zones were mainly observed as a function of exposure temperature and time. Some research has been conducted to minimize the width of these zones by using the advanced welding processes or to avoid the need of PWHT, which is the original cause of carbon migration zones formation [11,17,18,22,25]. Kulkarni et al. [11] investigated the effect of the Activated-Tungsten Inert Gas (A-TIG) welding process on the carbon diffusion distance and their effect on hardness variation along the interface. The average width of the carbon migration zones obtained for the A-TIG weld joint was lesser than the multi-pass welded joint of a similar combination of P22/P91 steel. The advanced joining process was also attempted to obtain an acceptable level of the mechanical properties for the welding joint of P91 and P22 steel in the AW condition. The width of the SZ and HZ and size of the HAZs were also measured to be lesser than the fusion welded joint, i.e., Gas Tungsten Arc Welding (GTAW) [25]. Sunil Kumar et al. [22] conducted a joining of P22 with 9Cr-1Mo steel using the Friction Stir Welding (FSW) process and the mechanical properties of the FSW joint were compared with the multi-pass GTAW joint. A study on carbon migration zones was also conducted for both GTAW and FSW joints. However, to avoid the necessity of the PWHT for such a type of joint, few studies relating to the use of Ni-based superalloy filler have been conducted. Kumar et al. [17] performed a joining of the P91/P22 steel using a Ni-based superalloy filler and reported the acceptable level of mechanical properties, i.e., impact toughness and tensile strength, for the welded joint in the AW condition. Tammasophon et al. [18] also conducted a microstructure and mechanical behavior study on a P22/P91 welds joint produced using the Inconel 625 filler. The optimum mechanical behavior was produced after PWHT; however, the study related to the diffusion of elements across the interface of P22 steel and IN625 weld metal was missing. 

From the literature survey, few studies have been found relating to the application of the dissimilar Ni-based superalloy filler. However, detailed characterization of the weld interface region and mechanical behavior study has not been reported yet for the Ni-based superalloy filler. The present work investigates the effect of the Ni-based superalloy filler ERNiCrMo-3 on the mechanical and microstructural behavior of the dissimilar GTAW joint of P91 and P22 steel. To study the formation of the carbon migration zones in ERNiCrMo-3 weld and P22 steel interface, PWHT was also performed at 760 °C for 2 h.

## 2. Materials and Methods

Two different grade Cr-Mo steel, i.e., ferritic/bainitic P22 (2.25Cr-1Mo) and ferritic/martensitic P91 (modified 9Cr-1Mo) of dimension 120 mm × 70 mm × 10 mm were used to prepare the GTAW joint. The Ni-based superalloy ERNiCrMo-3 was used as the filler material. The chemical composition of the base plates and filler metal are listed in Table 1.

**Figure 1:**
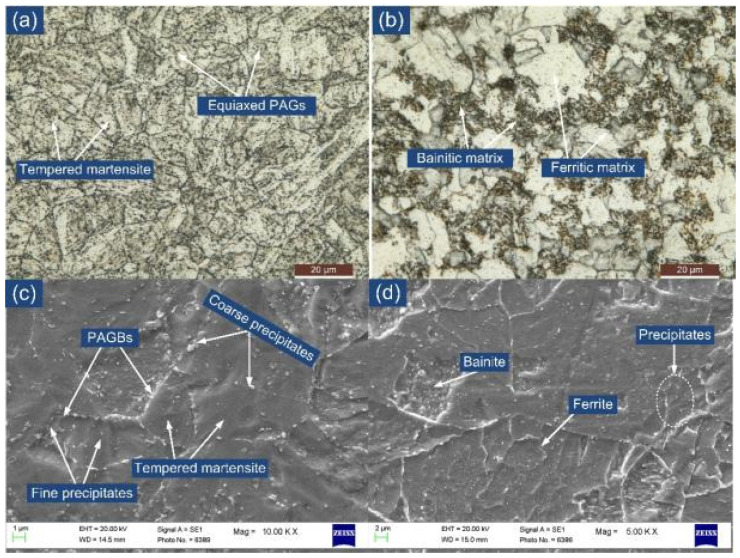
Typical optical image of (**a**) P22 steel, (**b**) P91 steel; SE image of (**c**) P22 steel, and (**d**) P91 steel.

The typical optical and secondary electron (SE) image of P91 and P22 steel is depicted in Figure 1a–d. A typical tempered martensitic matrix is seen for the P91 steel, as depicted in Figure 1a. A uniform distribution of the coarse precipitates along the boundaries and fine precipitates within the interior region of the matrix are seen from the SE image in Figure 1c. The coarse precipitates along the boundaries are confirmed as the Cr and Mo enriched M_23_C_6_ carbides, and their size is measured in a range of 100 to 250 nm. The fine spherical shape precipitates inside the interior region of the matrix are confirmed as V and Nb enriched MX type carbides and their size was reported in the range of 20 to 50 nm [26]. The tempered martensitic matrix shows the presence of the equiaxed prior austenite grains of average size 12 ± 4 μm. The grain size was measured using ImageJ software. The other precipitates in P91 steel were confirmed as M_7_C_3_ and M_6_C from XRD analysis [27]. A typical optical image of P22 steel exhibits the bainitic-ferritic microstructure having a random orientation of bainitic sheaths within the bainitic matrix Figure 1b. The optical image shows the equiaxed grains of average size 10 ± 4 μm. The SE image shows the distribution of the precipitates within the bainitic-ferritic matrix (Figure 1d). A large number of the globular shape precipitates can be noticed in the region of the bainite which are confirmed as Mo and Cr enriched M_23_C_6_ carbides of size 350 ± 45 nm and cementite stringer of average size 580 ± 60 nm. The precipitates are confirmed as M_23_C_6_, M_6_C, M_7_C_3_, and Mo_2_C from XRD analysis [11]. The tensile strength and impact toughness of the P91 steel are measured 715 ± 15 MPa and 252 ± 5 J [20,28], respectively. For P22 steel, tensile strength and impact toughness are 610 ± 2 MPa and 320 ± 8 J, respectively [20].

**Figure 2:**
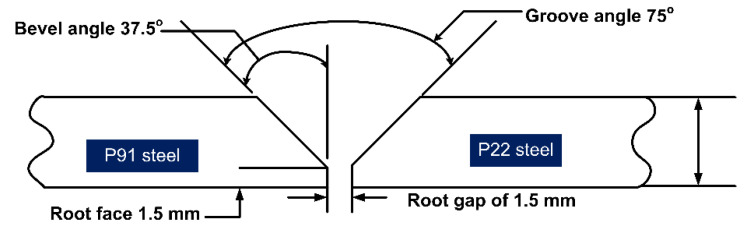
Conventional V-groove geometry.

A conventional V-groove (groove angle: 75°, root height: 1.5 mm, and root gap: 1.5 mm) has been machined at the edges of the plates as in Figure 2. The welding current and arc voltage used for the root pass and filling passes are listed in Table 2. The preheating was done at a temperature of 280 °C to minimize the problem related to hydrogen cracking while the inter-pass temperature was maintained by about 250 °C ± 10 °C using flame heating.

**Figure 3:**
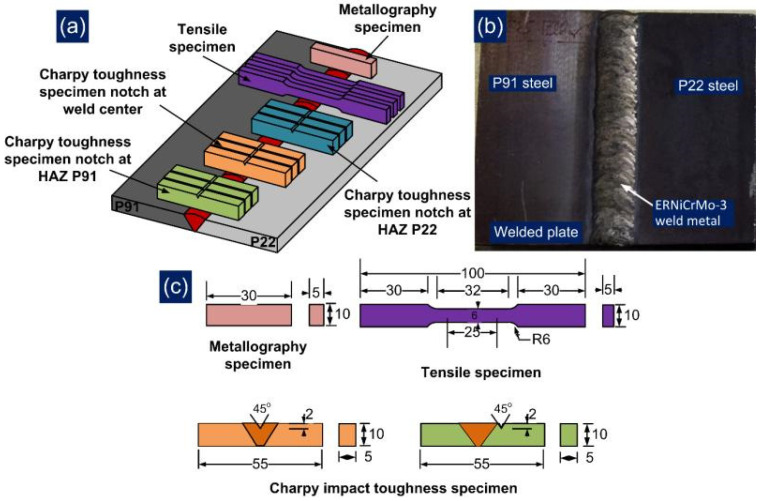
(**a**) Schematic image showing the extraction of the specimen from a welded plate, (**b**) view of welded plate, (**c**) dimensions of the testing specimens.

The welding was carried out in a shielding environment of pure Ar gas (SG-A) of purity 99.9%, which was supplied at the flow rate of 15 L/min. The PWHT of the welded joint has also been performed at 760 °C for 2 h. The mechanical testing specimen has been cross-sectioned from the welded plate using a wire cut EDM machine. The joint integrity has been tested by performing tensile testing, impact testing and hardness testing. The standard tensile specimen (Figure 3) has been prepared as per ASTM E8/E8M standards and the test was conducted on a vertical tensile testing machine (Instron 5980 of 100 kN capacity, Instron, MA, USA). The tensile test was done at room temperature with a constant strain rate of 6.66 × 10^−4^/s. The impact specimen of dimension 55 mm × 10 mm × 5 mm with a central V-notch of depth 2 mm was machined (Figure 3). The impact specimen was prepared for both weld metal and HAZ of P91 and P22 steel as per ASTM E23-13 and tested at room temperature on a Charpy impact tester (FIT-400-ASTM-D, Fine Testing Machines Pvt. Ltd., Miraj, India). The microhardness variation was measured along the transverse direction of the weldments, as given in Figure 3. The microhardness test was conducted at an interval of 0.5 mm for a constant load of 500 g using a microhardness tester (Mitutoyo, Model: Autovick HM-200, Mitutoyo, Kawasaki, Japan). For each tensile and impact testing condition, three specimens were machined from the welded plate to minimize errors and ensure repeatability in results. 

The sample for metallographic testing was cut and machined (Figure 3) and then subjected to a standard metallographic technique, i.e., grinding and polishing. The mirror-polished sample of the weldments was subjected to etching using Vilella’s solution for the HAZ region of P91 and P22 steel [29] and electro etching at 9 V in 10% oxalic acid solution for weld metal and the interface region [30]. The optical microscope (Leica, Model: DMC4500, Leica Microsystems GmbH, Wetzlar, Germany) and field emission scanning electron microscope (FESEM) equipped with energy-dispersive X-ray spectroscopy (EDS) (Carl Zeiss Ultra plus and FEI Quanta 200, Carl Zeiss Microscopy Deutschland GmbH, Oberkochen, Germany) were utilized for phase study of the weldments in both AW and PWHT condition. The residual stress measurement was also conducted in the weld metal region using blind hole drilling (BHD) methods. The other methods utilized for the residual stress measurement were X-ray diffraction (XRD), and deep hole drilling [31,32]. The measurement was performed along the thickness of the plate (at a depth of 2 mm from the top and root of the welded joint) to study the effect of the welding passes on residual stresses. The detailed steps used for residual stress measurement has already been discussed in a previous work [33].

## 3. Results and Discussion

### 3.1. Characterization of the Weldments

**Figure 4:**
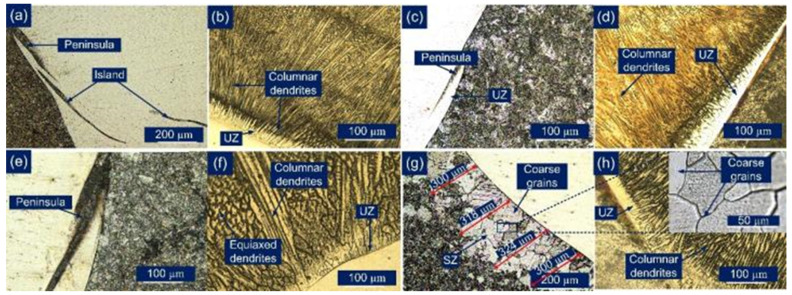
Optical image of the interface region in the AW joint: (**a**,**b**) interface of P91 steel and ERNiCrMo-3 weld metal; (**c**,**d**) interface of P22 steel and ERNiCrMo-3 weld metal; after PWHT: (**e**,**f**) interface of P91steel and ERNiCrMo-3 weld metal; (**g**,**h**) interface of P22 steel and ERNiCrMo-3 weld metal.

Figure 4 shows the microstructure of the weld metal and interface region of base material and ERNiCrMo-3 weld metal for the AW and PWHT joint. An unmixed zone (UZ) formation at the interface of weld metal and base material is inferred from Figure 4a–c. The UZ near the interface shows a peninsula and island-like structure, as depicted in Figure 4a–c. It is inferred from Figure 4a–c that the peninsula like microstructure is relatively homogeneous, distributing along the interface of the weld metal. However, a great gradient distribution is noticed for the island-like microstructure in Figure 4a. The columnar grain growth is noticed for the weld metal near the interface, as given in Figure 4b–d. The steeper thermal gradient present in the fusion boundary over the bulk weld metal allows the grain to grow in the direction opposite to heat dissipation. This results in grain growth near the edges of the weld pool that is observed towards the weld center as in Figure 4a,b. The grain growth is referred to as the columnar grain growth, and the results obtained are well in agreement with previously published work [34,35]. Figure 4e–h shows the effect of the PWHT on the microstructural features of the interface as well as the weld metal near the interface. The PWHT was found to have a negligible effect on the microstructural behavior of the interface region of the P91 steel and ERNiCrMo-3 weld metal as in Figure 4e. The austenitic microstructure in the weld metal near the interface also shows a poor response to PWHT, as noticed in Figure 4f. However, a significant change in grain structure occurs at the interface of the P22 steel and ERNiCrMo-3 weld metal. The weld metal shown in Figure 4h near the interface remains unaffected; however, along the P22 side, a coarse grain structure is observed, as marked in Figure 4g,h. This region is inferred to as a soft zone (SZ), which forms mainly due to carbon migration from the P22 steel to ERNiCrMo-3 weld metal. The grain size in SZ was measured. A similar result, i.e., C migration at the interface of P22 steel and weld metal with high Cr content, has also been reported in previous studies [3,12,15,20]. The detailed mechanism of SZ formation has been discussed by Sudha et al. [14] and Pandey et al. [36]. In the AW condition, there is no evidence of SZ formation or C migration, as given in Figure 4c. However, after the PWHT, clear evidence of SZ formation is found in Figure 4g, and from current observations, it can be inferred that the C diffusion at the interface is a mainly temperature-controlled mechanism. The average width of the SZ is measured at 310 µm. The C migration at interface also leads to the formation of the HZ adjacent to SZ, and it occurs mainly due to the formation of the diffused C with Cr present in ERNiCrMo-3 weld metal.

**Figure 5:**
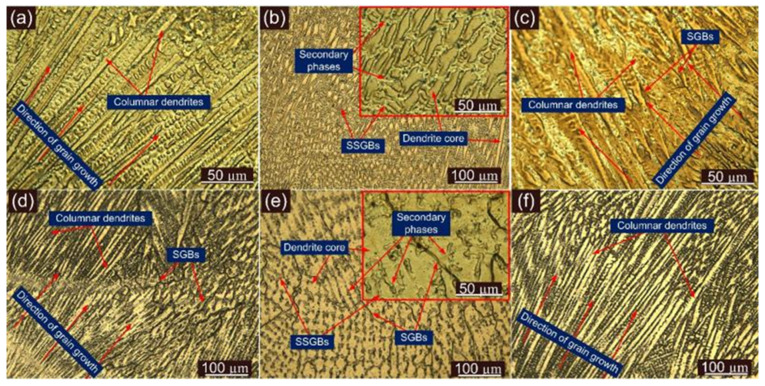
Typical optical micrograph of the ERNiCrMo-3 weld metal near the interface and in the interior region for AW joint: (**a**) ERNiCrMo-3 weld metal near P91 interface; (**b**) interior region of the bulk weld metal, (**c**) near P22 interface; after PWHT: (**d**) ERNiCrMo-3 weld metal near P91 interface, (**e**) interior region of the bulk weld, (**f**) near P22 interface.

Figure 5 depicts the microstructure of the ERNiCrMo-3 weld metal near the interface of P22 steel, P91 steel, and at the interior region for both AW and PWHT conditions. It is inferred from Figure 5a–c that columnar dendrites are predominant at the interface area for both sides while the weld center exhibits the equiaxed dendrites shown in Figure 5b. The steep thermal gradient at the interface allows the grain to grow in a direction opposite to heat dissipation and results in grain growth from the edge of the weld pool to the weld center as in Figure 5a–c. The grain growth is referred to as the columnar grain growth, and the growth direction is also marked in Figure 5a–c. The equiaxed dendrites in the weld center for Ni-based superalloy filler with GTAW process have also been reported in previous research [20,37,38,39,40,41], and the presence of a high level of super constitutional cooling during solidification in the GTAW process has been mainly reported as an important factor which governs the formation of the equiaxed dendrites in the weld center [42]. The microstructure results obtained at the interface and in the weld center are well in agreement with previously published work by Chandrasekar et al. [34] and Ramkumar et al. [43]. The inter-dendritic areas show the segregation of the alloying elements as referred from Figure 5b. After the PWHT, the interface region of ERNiCrMo-3 weld metal shows a negligible change, and it occurs mainly due to the negligible response by the austenitic microstructure of weld metal to PWHT. However, solidified grain boundaries (SGBs) appear both at the interface and interior region of the weld metal as a result of PWHT as in Figure 5d,e. The interior weld metal shows the typical austenitic microstructure having equiaxed dendrites as in Figure 5d. The boundaries (SSGBs and SGBs), dendrite core and segregation of the alloying elements are marked in Figure 5d.

**Figure 6:**
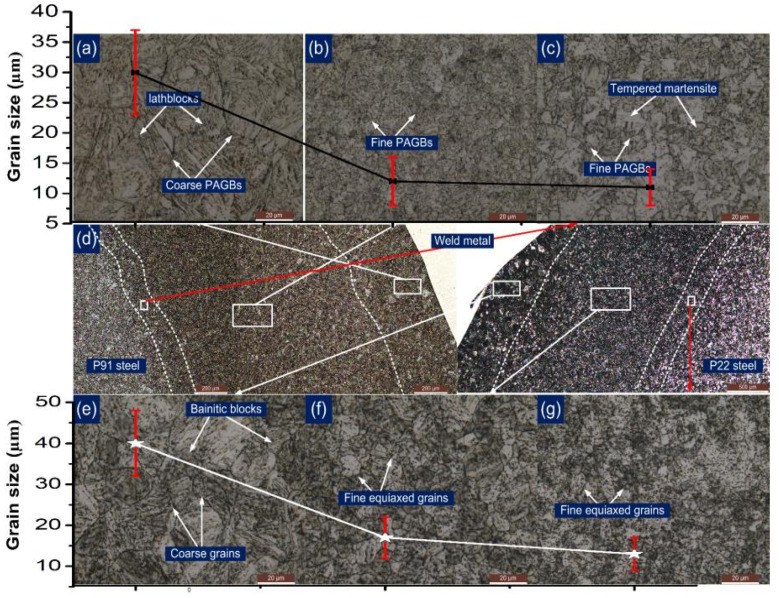
Inhomogeneity in microstructure along the P91 HAZ after welding: (**a**) coarse-grained HAZ, (**b**) fine-grained HAZ, (**c**) inter-critical HAZ; (**d**) optical image showing the various HAZ regions formed along P91 and P22 side; P22 HAZ region after welding: (**e**) coarse-grained HAZ, (**f**) fine-grained HAZ, (**g**) inter-critical HAZ.

An inhomogeneity in microstructure along the P91 and P22 HAZs is inferred clearly from Figure 6. Figure 6d shows the formation of the wide HAZ region on both sides of the ERNiCrMo-3 weld metal. The evolution of the gradient microstructure in the HAZ region of P91 and P22 steel depends on the peak temperature experienced during the welding cycle [29]. The coarse-grain microstructure consists of untempered martensite with lath block, is noticed in the HAZ region of P91 near the weld fusion boundary and named as coarse-grained HAZ (CGHAZ) as in Figure 6a. The grains in HAZ get coarsened because of the high peak temperature (˃˃A_c3_), which causes the dissolution of the inherent precipitates present along the grain boundaries in as-received material shown in Figure 1b. The precipitates located along the grain boundaries mainly restrict the movement and provide the pinning effect. It is inferred from Figure 6b that grain boundaries are smaller in size in the region next to CGHAZ and named as fine-grained HAZ (FGHAZ). This experiences a temperature near to A_c3_ but with a retention time lower than the CGHAZ, which allows a partial dissolution of the precipitates and results in restricted coarsening in FGHAZ. However, the temperature nearer to A_c3_ causes a coarsening of the undissolved carbide precipitates. The recrystallization in FGHAZ results in the formation of new PAGs which are pinned by the undissolved precipitates. The region of HAZ adjacent to FGHAZ on base metal side experiences temperatures between A_c1_ and A_c3_ which results in a partial transformation of the austenite which are transformed into fresh martensite after cooling. However, the rest of the region shows the overtempering of the martensite and its causes a complex microstructure of fresh martensite (austenitic transformation products) and tempered martensite (untransformed ferrite) exists in the region. The region is named as inter-critical HAZ (ICHAZ) Figure 6c. The variation in grain size along the HAZ region of P91 steel is also marked in Figure 6a–c.

A similar trend of microstructure variation is also observed on P22 side HAZ, as mention in Figure 6e–g. The region of P22 CGHAZ near the fusion boundary shows the coarse bainitic microstructure (Figure 6e). It is inferred from Figure 6f that P22 FGHAZ has a bainitic microstructure, however the size of the grains is measured to be lower than those in the CGHAZ. The region of P22 ICHAZ exhibits a complex microstructure of tempered bainite (untransformed ferrite) and austenitic transformation products (ATP) as in Figure 6g. The variation in grain size along the HAZ region of P22 steel is marked in Figure 6e–g.

**Figure 7:**
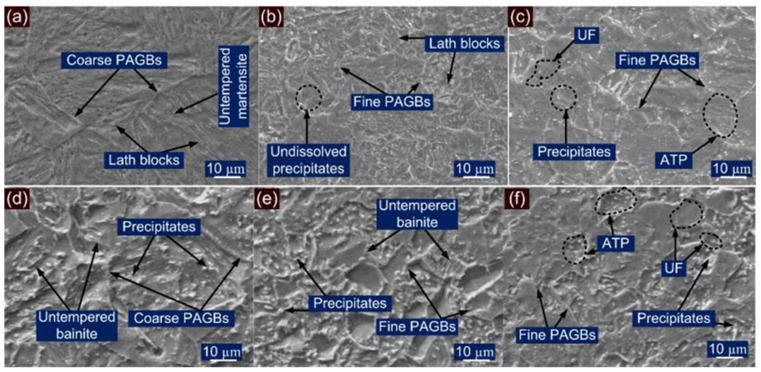
Typical SE image of HAZ region of P91 steel: (**a**) coarse-grained HAZ, (**b**) fine-grained HAZ, (**c**) inter-critical HAZ; HAZ region of P22 steel: (**d**) coarse-grained HAZ, (**e**) fine-grained HAZ, (**f**) inter-critical HAZ.

The SEM observation is also made for the HAZ region of P91 and P22 steel and presented in Figure 7. A typical lath block-like structure is revealed in the region of CGHAZ as shown in Figure 7a. The CGHAZ exhibits an untempered martensitic microstructure with coarse PAGBs. FGHAZ shows the fine PAGBs and undissolved coarse carbide precipitates as in Figure 7b. The undissolved precipitates in FGHAZ were mainly confirmed as Cr and Mo enriched M_23_C_6_ carbides [11]. The higher thermal stability of the fine MX precipitates as compared with M_23_C_6_ has been reported in the literature, and MX precipitates in FGHAZ remain undissolved [28,44]. The size of the undissolved precipitates is measured at 150 ± 45 nm. The ICHAZ shows the UF and ATP as in Figure 7c. The density of the precipitates in ICHAZ is also measured to be higher than in FGHAZ, mainly due to low peak temperature in ICHAZ, which causes negligible dissolution of the precipitates. The size of the precipitates in ICHAZ is also measured to be higher than in the FGHAZ and the average size measured at 165 ± 56 nm. The SEM observation of the P22 HAZs also shows the dissimilarity in microstructural behavior. The region of the P22 CGHAZ in Figure 7d exhibits a large density of undissolved precipitates inside the bainitic ground which were confirmed as cementite stringer (Fe_3_C), M_23_C_6_ and Mo_2_C [11] through XRD analysis. The coarse undissolved precipitates in the matrix of the bainite is also observed for FGHAZ/ICHAZ of P22 steel in Figure 7e,f. From Figure 7e, it is revealed that FGHAZ exhibits an untempered bainitic microstructure with fine PAGBs. The P22 ICHAZ does not show any major phase change and after cooling exhibits untransformed ferrite (tempered bainite) and ATP. The size of the undissolved precipitates is measured to be 415 ± 45 nm in the region of FGHAZ/ICHAZ.

**Figure 8:**
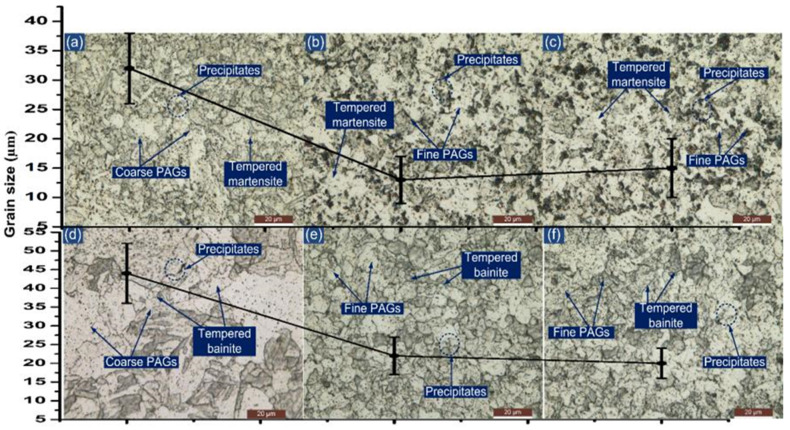
Typical optical image of HAZ region of P91 steel after PWHT: (**a**) coarse-grained HAZ, (**b**) fine-grained HAZ, (**c**) inter-critical HAZ; HAZ region of P22 steel: (**d**) coarse-grained HAZ, (**e**) fine-grained HAZ, (**f**) inter-critical HAZ.

PWHT had a positive effect on the microstructural behavior of the HAZ region, as presented in Figure 8. However, the grain size gradient still exists on both sides of the HAZ region as in Figure 8. Minute grain coarsening occurs in each region of the HAZ after the PWHT and can be disregarded. After the PWHT, a typical tempered martensitic microstructure is formed in each region of P91 HAZ, as shown in Figure 8a–c. A tempered bainitic microstructure is noticed in each HAZ region of P22 steel as can be seen from Figure 8d–f.

**Figure 9:**
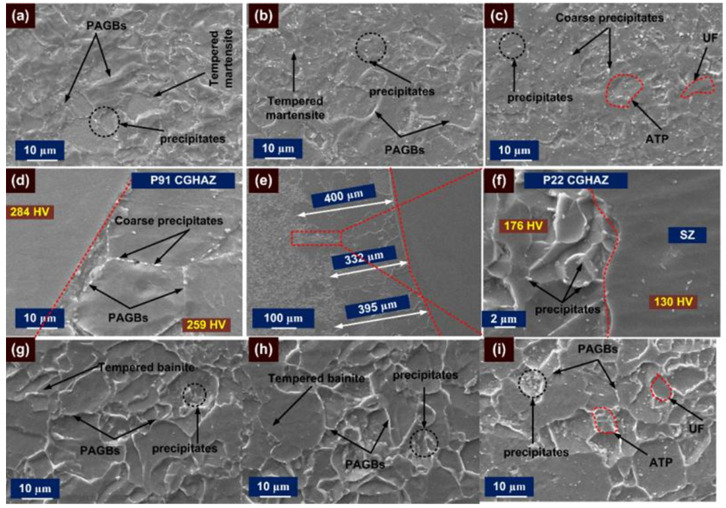
Typical SE image of HAZ region of P91 steel after PWHT: (**a**) coarse-grained HAZ, (**b**) fine-grained HAZ, (**c**) inter-critical HAZ; interface region: (**d**) on P91 side, (**e,f**) on P22 side; HAZ region of P22 steel: (**g**) coarse-grained HAZ, (**h**) fine-grained HAZ, (**i**) inter-critical HAZ.

An SE image of the HAZ region and interface after PWHT is presented in Figure 9. The CGHAZ region of P91 steel exhibits a tempered martensitic microstructure having both coarse carbide and fine carbonitride precipitates (Figure 9a). It is revealed in Figure 9d that CGHAZ near the interface shows a decoration of carbide precipitates along PAGBs, which are larger in size than the CGHAZ region away from the interface (Figure 9a). The hardness gradient also exists near the interface as marked in Figure 9d. The average size of the precipitates was measured at 176 ± 45 nm, which was less than the FGHAZ/ICHAZ. Both FGHAZ in Figure 9b and ICHAZ in Figure 9c show a tempered martensitic microstructure with a distribution of coarse carbide precipitates along the boundaries and fine precipitates within the matrix. The average size of the precipitates was measured at 180 ± 42 nm and 205 ± 52 nm for FGHAZ and ICHAZ, respectively. However, ICHAZ still shows a softening nature due to the presence of ATP and over-tempered UF as in Figure 9c. The softening of ICHAZ is also confirmed through the hardness results as presented in a later section. 

The CGHAZ in Figure 9g and FGHAZ in Figure 9h regions of P22 steel exhibit a tempered bainitic microstructure with a great difference in grain size as shown in optical image. The bainitic region of P22 CGHAZ shown in Figure 9f shows precipitates of acicular and globular morphology, which were detected as the carbide of type M_23_C_6_ and Fe Cr and Mo enriched precipitates [11]. The average size of the globular shape precipitates was 250 ± 45 nm, while the length of the acicular shape particle was 510 ± 35 nm. The region of FGHAZ also shows globular shape precipitates of size 215 ± 52 nm and the acicular shape precipitates of size 680 ± 182 nm. In the tempered ICHAZ region, precipitates are observed both in the bainitic matrix and ferritic matrix as in Figure 9i. The precipitate size in ICHAZ was measured at 300 ± 58 nm, which were confirmed as the carbide precipitates of types M_23_C_6_ and M_6_C [11]. In AW conditions, there was no evidence related to the SZ and HZ at the interface of P22 steel and ERNiCrMo-3 weld metal as shown in Figure 4c. However, SZ formation is seen clearly near the interface of the P22 steel and ERNiCrMo-3 weld metal as the cause of PWHT as in Figure 4g. The interface is characterized further using the SE image presented in Figure 9e,f. In the P22 steel side, a coarse grain structure formation is noticed in a soft zone as in Figure 9e, and width is measured 376 µm. However, from the optical image width was measured at 310 µm. The detailed view is referred in Figure 9 and shows the interface of the SZ and HAZ region of P22 steel. A negligible amount of the precipitates is observed in the SZ, while in the P22 HAZ, coarse precipitates are higher in the bainitic matrix. The hardness gradient also exists at the interface of the SZ and HAZ as presented in Figure 9f. 

The formation of the SZ and HZ near the interface of P22 steel and high Cr content weld metal is a common phenomenon that occurs due to the diffusion of the C [15,45] during PWHT. At the interface of P91 steel and ERNiCrMo-3 weld metal, a diffusionless transformation occurs due to the negligible variation in carbon activity; however, at the P22 steel and ERNiCrMo-3 weld metal interface, a variation in carbon activity leads to the formation of carbon migration zones, i.e., SZ and HZ. In the AW joint, a negligible carbon migration was reported by Sudha et al. [14], which results in the absence of the SZ and HZ in the AW joint. However, the diffusion of the C during PWHT reflects the formation of a continuous band of coarse grains near the interface of weld metal and P22 steel, which shows negligible precipitates and offers poor hardness compared with the rest of the weldments (130 HV) shown in Figure 9f. The detailed mechanism of SZ formation has been discussed by Pandey et al. [36]. The diffused carbon from the P22 steel gets combined with the Mo and Cr present in the ERNiCrMo-3 weld metal and results in the evolution of the Cr- and Mo-enriched carbides of type M_6_C and M_23_C_6,_ which increase the hardness in this zone, i.e., hard zone (HZ). The diffusion of C from the P22 steel side is reflected in terms of the dissolution of ferrite stringer of higher C content which leads to the formation of coarse grains of pro-eutectoid ferrite (SZ) of low hardness. Compared with the SZ, the HZ’s growth rate was negligible and the size was also much smaller; however, SZ and HZ were detected clearly in hardness variation. The formation of the SZ and HZ had also been demonstrated in a previous published work on dissimilar joining of P22/P91 steel. The width of the carbon migration zone was reported to be higher in A-TIG and multi-pass TIG welding process with matching P91 filler [11,15,20]; however, a narrow width was measured for the laser beam welded joint [25]. The formation of carbon migration zones during the PWHT mainly affects the creep and fatigue properties of the welded joint [10,45].

**Figure 10:**
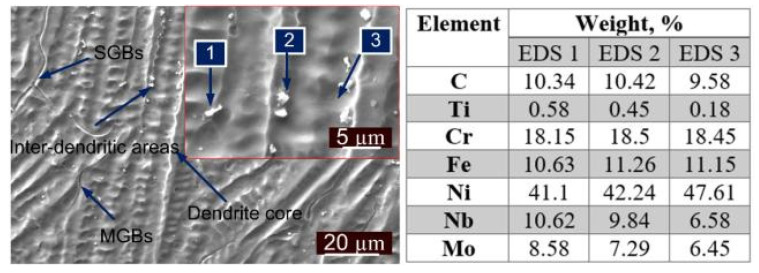
Weld metal near the interface and EDS spectrum corresponding to the marked point.

**Figure 11:**
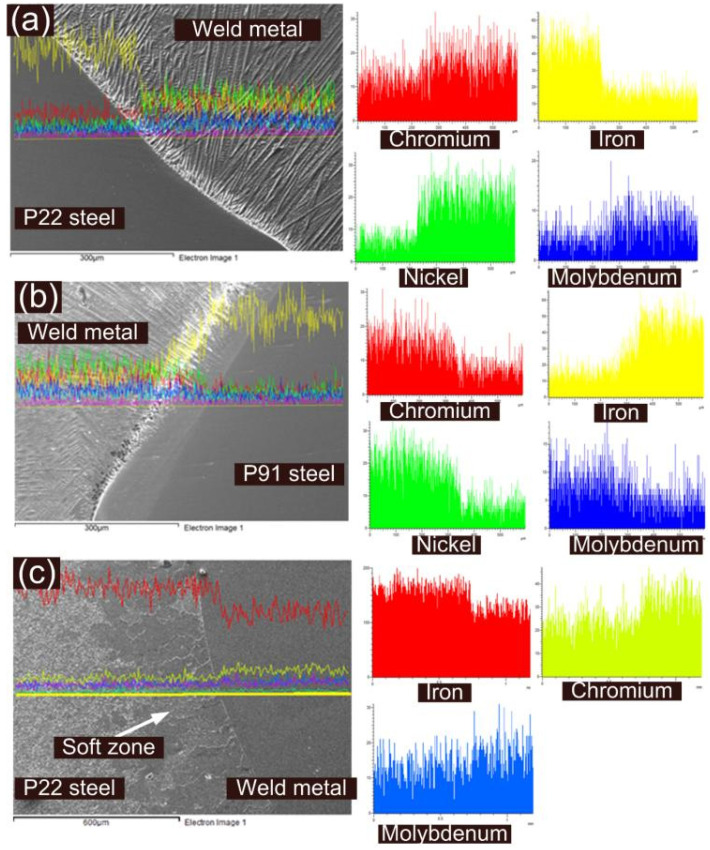
Elemental profile at the interface of (**a**) P22 steel and ERNiCrMo-3 weld metal, (**b**) P91 steel and ERNiCrMo-3 weld metal for AW joint; (**c**) P22 steel and ERNiCrMo-3 weld metal after the PWHT.

The weld metal near the interface is characterized at higher magnification for the AW joint and shows columnar grain growth along with segregated particles in inter-dendritic areas, which are confirmed as Nb- and Mo-enriched carbides or Laves phase by EDS spectrum (EDS 1 and EDS 2: Figure 10). From EDS results of the inter-dendritic areas (EDS 3: Figure 10), major segregation is observed for the Nb and Mo. The higher carbon content might be due to the carbide phase. Figure 11 shows the distribution of the Ni, Cr, Fe, Mo, Nb and Ti across the interface of base metals (P22 and P91) and ERNiCrMo-3 weld metal. From Figure 11a, it is inferred that Ni and Cr diffused to P22 steel from the ERNiCrMo-3 weld metal while Fe diffused into ERNiCrMo-3 weld metal from P22 steel. A similar observation is also made for the P91 interface (Figure 11b). The interface of P22 steel and ERNiCrMo-3 weld metal after PWHT is delineated in Figure 11c.

**Figure 12:**
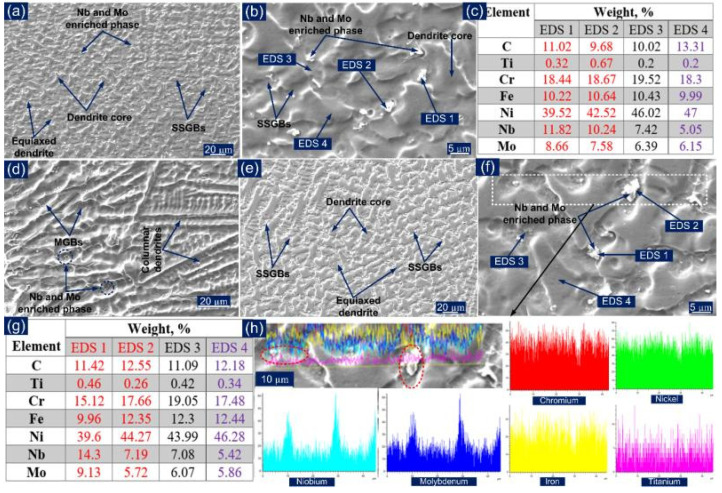
(**a**) AW joint: SE image of the weld metal showing the equiaxed dendritic microstructure, (**b**) marked EDS location, (**c**) EDS spectrum results; PWHT: (**d**) near interface showing columnar dendrites, (**e**) interior weld metal showing equiaxed dendrites, (**f**) marked EDS location, (**g**) EDS spectrum results; (**h**) line mapping across the inter-dendritic boundaries in the region of bulk weld metal.

Like P91 filler, Ni-based filler does not show the solid-state phase transformation to get the final welds. Hence, grain structure, i.e., size and shape, segregation at the micro and macro level, defects like hot crack, inclusion and porosity, and mechanical properties of the welded joint, is mainly controlled by the solidification behavior. The weld metal solidifies as a Ni-rich austenite phase with equiaxed dendrites, as shown in Figure 12a. For the ERNiCrMo-3 filler, the mode of solidification is given by Equation (1) [46], where filler solidifies first as a final microstructure of Ni-enriched austenite with precipitation of the Nb-enriched NbC and Laves phase in the inter-dendritic areas, as depicted in Figure 12b.

(1) L→L+γ→L+γ+NbC→L+γ+NbC+Laves→γ+NbC+Laves

The low thermal conductivity of the ERNiCrMo-3 welds, mainly due to the high alloying level, leads to the high-temperature gradient in the weld metal. The solidification mode in ERNiCrMo-3 weld is also influenced by the degree of the cooling rate (°C/s) which is given by Equation (2) [46];

(2) ε=G×R

where G is the temperature gradient (°C/m) in the liquid, R denotes the solidification front (or crystal) growth rate (µm/s).

It can be inferred from Equation (2) that dendrite spacing depends on the cooling rate and increases with an increase in the cooling time, i.e., with an increase in heat input welding. 

The other parameter which affects the dendrite morphology of solidified microstructure is constitutional supercooling. David et al. had expressed a mathematical expression to define the criterion for constitutional supercooling for plane front instability given in Equations (3) and (4);

The plane front will be stable when:

(3) $$\frac{G}{R}\geq \frac{∆T_{0}}{D_{L}}$$

Planer instability will occur when:

(4) $$\frac{G}{R}<\frac{∆T_{0}}{D_{L}}$$

where $∆T_{0}$ is the equilibrium solidification temperature range (at composition C_0_) and D_L_ is solute diffusion coefficient (m^2^/s) in liquid.

The G·R ratio controls the solidification mode, while the size of the structure is controlled by the G·R. A higher G·R ratio occurred at the fusion boundary and decreased as it moved from the fusion boundary to the bulk weld metal while the degree of constitutional supercooling increased, resulting in the transformation of the solidification mode from columnar and cellular as in Figure 12d to equiaxed dendrites as in Figure 12.

The variation in microstructure is also observed at the micro level due to segregation of the alloying elements (Fe, Cr, Nb. Mo) at the inter-dendritic boundaries during the solidification process. The high magnification SE image of the center weld metal in Figure 12b shows the segregation of the alloying elements at the inter-dendritic areas, leading to secondary phase formations like the NbC and Laves phase. The composition analysis in Figure 12c shows that the dendrite core consists of Ni, Cr and Fe (EDS 4) while inter-dendritic areas include Nb, and M (EDS 1, EDS 2 and EDS 3). The EDS spectrum results of the white precipitates (EDS 1 and EDS 2) also show the major weight percentage of Nb and Mo, which confirms the precipitation of the Nb-enriched phase NbC and Laves and Mo-enriched phase Mo_2_C and Laves [47]. The Laves phase is an intermetallic compound of type M_2_X (M: Ni, Cr; X: Mo or Nb).

The weld metal near the interface and in the interior region is depicted in Figure 12d–f for the PWHT joint. The columnar dendrites at the P91 steel and weld metal interface in Figure 12d and the equiaxed dendrites in the center of the weld metal in Figure 12e are noticed, which shows that the scale of the dendritic microstructure is variable as with the AW joint. The interface region of the weld metal also shows the segregation of the alloying elements along the inter-dendritic boundaries as in Figure 12d. The region also shows the migrated grain boundaries (MGBs). The center region shows equiaxed dendrites as observed in the AW condition as in Figure 12e. The higher magnification image was selected for the EDS analysis, as presented in Figure 12f. The EDS spectrum results show a similar observation as obtained for the AW joint. The major segregation of Ni, Cr and Fe was observed in the dendrite core, while inter-dendritic areas show the segregation of the Nb and Mo as in Figure 12g. The composition analysis of the white precipitates ensured the presence of the Nb- and Mo-enriched secondary phases in the inter-dendritic areas shown in Figure 12g. A small region was selected from the center region of the weld metal to perform the elemental line mapping, which covers the inter-dendritic boundaries and dendrite core. The line mapping also ensures the major segregation of the Nb and Mo at the inter-dendritic boundaries as in Figure 12h. The higher peak density of Nb and Mo is observed for the white precipitates as shown in Figure 12h. The formation of the Nb enriched NbC and Laves phase in the inter-dendritic areas of the weld metal is mainly associated with the extensive segregation of the Nb and C during solidification. Furthermore, Ramkumar et al. [48] have reported that the poor solubility of Mo in the austenitic matrix due to the large size radius results in segregation of Mo at the inter-dendritic areas as observed from Figure 12c,g, which facilitates the formation of Mo-enriched Mo_2_C or Laves phase. The formation of the Mo- and Cr-enriched M_23_C_6_ phase in the weld metal region has also been reported in previous research [49,50,51].

### 3.2. Tensile Properties

**Figure 13:**
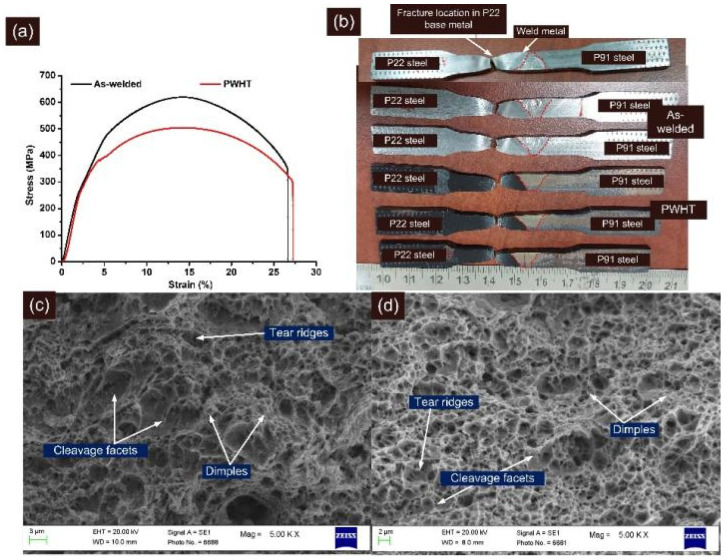
(**a**) Stress–strain curve for tensile tested specimen, (**b**) fractured tensile specimen, (**c**) fractured surface image for AW joint, (**d**) PWHT joint.

The tensile test results are depicted in Table 3. The stress–strain curve is shown in Figure 13a. The fractured tensile specimen is shown in Figure 13b, and it is inferred that the fracture occurs in a ductile manner, i.e., cup-cone fracture for both AW and PWHT tested specimens. The fracture was noticed in the P22 steel base metal region instead of the weld metal, which ensures that the welded joint is safe for application in super-critical power plants.

For the AW joint, yield strength and tensile strength were 415 ± 5 MPa and 615 ± 4 MPa, respectively, which was close to the P22 base metal but lower than the P91 steel. The % elongation was also measured at 26.67 ± 0.57% which was in between P91 and P22 steel. Previous studies related to the dissimilar joint of P91 and P22 steel have also showed a failure in the region of P22 base metal and the strength of the welded joint was also measured near to the strength of P22 base metal [17,20]. For P22/P91 welded joint with matching P91 filler, strength was measured at 611 ± 9 MPa [20], while for IN617 filler, it was 618 ± 30 MPa [17]. For the A-TIG weld joint, strength was measured at 522 MPa by Kulkarni et al. [11]. The joint efficiency was measured at 86% for the AW joint due to the fracture from the region of P22 base metal. The PWHT of the welded joint reduced the tensile and yield strength with a minute increase in the % elongation. The yield strength and tensile strength were measured at 382 ± 5 MPa and 493 ± 11 MPa, respectively for the PWHT joint. The reduction in strength and increase in ductility might be due to the over-tempering of the bainite. The fractures in the PWHT joint were still observed in the P22 base metal region as shown in Figure 13b, which was far away from the soft zone, confirming that the carbon migration zones formed at the interface of P22 steel and weld metal did not affect the tensile properties of the welded joint. A similar observation has also been made by Kulkarni et al. [11]. However, for IN617 filler [17] and P91 filler [20], the welded joint strength after the PWHT was measured to be more than the strength of the P22 base metal, and fracture location was also observed in the region of P22 HAZ. The reduction in joint efficiency was also observed after the PWHT, and it was measured at 69%.

The FESEM fractographs of the tensile tested specimen are shown in Figure 13c,d. Figure 13c,d revealed a major presence of dimples, which implied the ductile mode of the fracture for both conditions. The cup-cone structure formation observed from Figure 13b also confirmed the ductile mode of the fracture. However, few small blotches of the cleavage facets, tear ridges, and elongated dimples of varying size and shape were also observed.

### 3.3. Microhardness Variation

**Figure 14:**
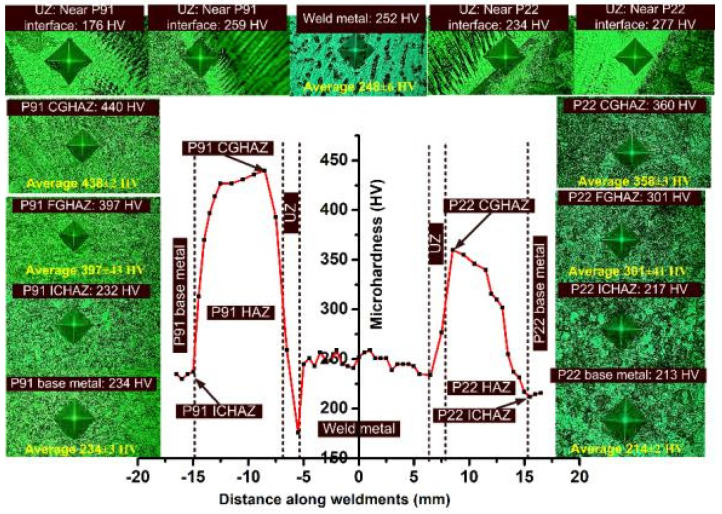
Hardness profile along P22/P91 dissimilar weldments for AW joint.

The hardness test was conducted on GTAW dissimilar weldments, and the results are shown in Figure 14. The microhardness indentation marks are also depicted in Figure 14. The indentation marks and hardness plots clearly show that the hardness of the weld metal had plummeted compared with the CGHAZ and FGHAZ region of the weldments. The average hardness of the weld metal was measured at 248 ± 6 HV. A small variation in the hardness value was observed for the weld metal, and the variation was attributed to the inhomogeneity in the microstructure of the weld metal, i.e., cellular, columnar, and dendritic along with segregated alloying elements. The UZ near the interface of P91 steel and weld metal showed a hardness of 259 HV, which was higher than the average hardness value of the weld metal but lower than the hardness of CGHAZ/FGHAZ. The peninsula formed along the interface shows a hardness of 176 HV, which was the minimum for the welded joint. The hardness of the P91 CGHAZ near interface is 440 HV which was very high and generates the necessity of the PWHT after the completion of the welding. The use of the welded joint with such a hardness value of CGHAZ can have a detrimental effect, such as hydrogen-induced cracking (HIC). Near the interface of P91 base and weld metal, i.e., in a small region, a significant level of heterogeneity in hardness value existed and it varied from 176 HV (peninsula) to 438 ± 2 HV (P91 CGHAZ). The higher hardness of the P91 CGHAZ was due to untempered lath martensite with a high weight percentage of C and N in the solution matrix, as shown in Figure 7a. The region of the FGHAZ was wide and a significant level of hardness variation was observed. The average hardness in P91 FGHAZ was 397 ± 43 HV. The hardness of the P91 ICHAZ and base metal was 232 HV and 234 ± 3 HV, respectively and the lower hardness in ICHAZ/base metal can be attributed to a tempered martensitic microstructure and coarse carbide precipitates. A variation in hardness also existed at the interface of the P22 and weld metal. The minimum hardness of 234 HV was measured at the interface, while in the peninsula and P22 CGHAZ it was 277 HV and 358 ± 3 HV, respectively. The hardness of the P22 CGHAZ was measured lower than the hardness of P91 CGHAZ and it might be due to its poor hardenability. The hard and brittle microstructure in P22/P91 CGHAZ with hydrogen can result in HIC. The untempered bainitic microstructure in P22 CGHAZ is attributed to the higher hardness value. Like P91 FGHAZ, P22 FGHAZ was also a wide region and showed a significant level of hardness along with an average hardness value of 301 ± 41 HV. The ICHAZ and P22 base metal showed hardness of 217 and 214 ± 2 HV. In the AW joint, the presence of the soft region like UZ (176 HV near P91 interface and 277 HV near the P22 interface), and ICHAZ (217 HV for P22 ICHAZ and 232 HV for P91 ICHAZ) acts as the cited stress raiser and might be responsible for the mechanical performance of the dissimilar welded joint of P22/P91 steel.

**Figure 15:**
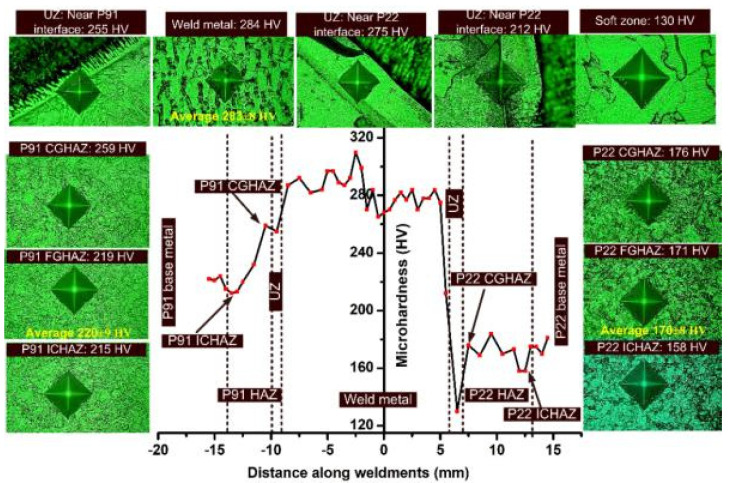
Hardness profile along P22/P91 dissimilar weldments after PWHT.

To provide the softening to CGHAZ of P91 and P22, tempering was done at 760 °C for 2 h, and the hardness profile obtained along the weldments is depicted in Figure 15. In the HAZ of P91 and P22 steel, the PWHT resulted in a drastic reduction in hardness; however, heterogeneity still existed along the weldments. The tempering of the martensite in CGHAZ of P91 resulted in a reduction in hardness value from 438 ± 2 HV to 259 HV, and in similar fashion bainite tempering in P22 CGHAZ reduced the hardness from 358 ± 3 to 176 HV. The interface of both steels with the base metal still showed a hardness variation. At the interface of the P91 steel and weld metal, hardness was 255 HV. The PWHT resulted in the formation of an SZ near the interface of P22 steel and weld metal (as discussed in Figure 9e, which shows the hardness of 130 HV). The UZ near the weld metal and near the soft zone of P22 steel showed a hardness of 275 HV and 212 HV, respectively. The high hardness of 275 HV near the weld metal side might be due to the carbon-enriched HZ. The reduction in hardness of the FGHAZ of P91 and P22 steel was also measured. The hardness of the P91 FGHAZ was 220 ± 9 HV which was attributed to its tempered martensite microstructure. In P22 FGHAZ, tempering of the bainite was also attributed to the reduction in hardness from 301 ± 41 HV to 170 ± 8 HV. The overtempering of the martensite in ICHAZ caused a reduction in hardness from 232 to 215 HV. A similar observation was also made for the region of P22 ICHAZ as a result of the over-tempering of the bainite. The minimum hardness after PWHT was 130 HV in SZ, while the maximum was measured in the weld metal, which was 283 ± 8 HV. The increase in hardness of the weld metal was observed after the PWHT. The increase in hardness might be due to precipitation of the gamma prime (γ′, Ni_3_NbAlTi) in the austenitic matrix of the weld metal. The precipitation of Nb- and Mo-enriched Laves phase also increased the hardness of the weld metal. A similar observation has also been made by previous researchers [18,53].

### 3.4. Charpy Impact Toughness

**Figure 16:**
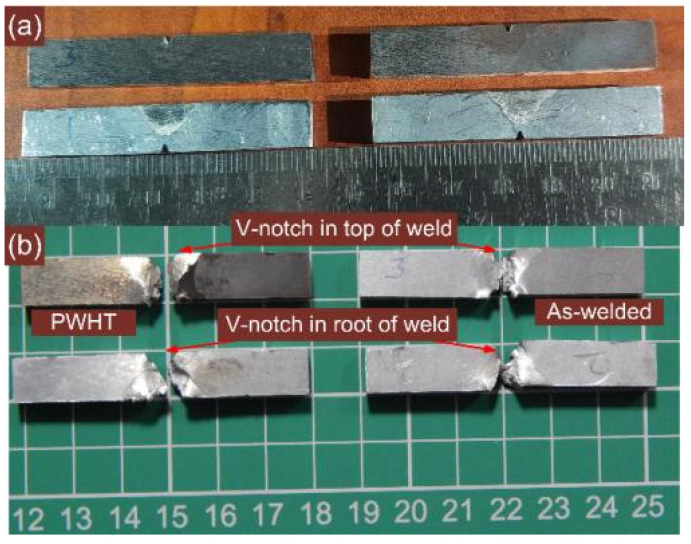
(**a**) Position of notch in top and bottom portion, (**b**) fractured impact toughness specimen for the weld metal.

**Figure 17:**
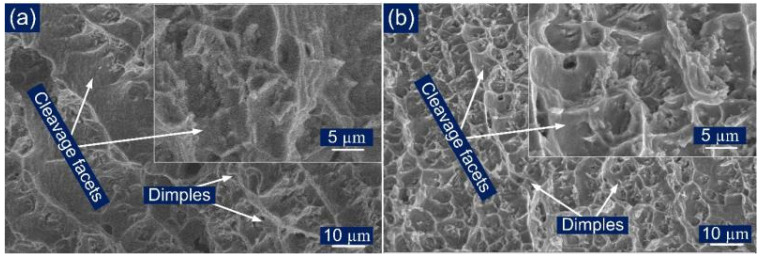
Typical fracture surfaces of the impact tested specimen for weld metal (**a**) AW, (**b**) PWHT.

The impact toughness test was conducted to ascertain the performance of the dissimilar weldments to sudden impact loading. The notch was made both in the top and root portion of the weld metal, as shown in Figure 16. The tests were also conducted for the HAZ region of P91 and P22 steel and to serve this purpose a notch was cut in the HAZ region. The test results are given in Table 4. From Table 4 it can be inferred that, as with hardness variation, impact toughness also varies significantly along the weldments. It is shown in Figure 16b that samples had undergone complete fracture rather than plastic deformation for each condition of the weld metal. The impact strength was 65 ± 3 J for the top weld in the AW joint and was reduced after the PWHT (58 ± 4 J). The fracture surface shown in Figure 17a,b also supports the impact test results. In the root impact test of the weld metal, impact strength was measured 74 ± 3 J and 69 ± 5 J for AW and PWHT joint, respectively. The impact strength of the weld metal in the top and root test was measured lower than the impact strength value of P91 base metal (105 J) and P22 base metal (188 J). The poor impact strength of the weld metal might be due to the segregation of the alloying elements in the region of inter-dendritic boundaries [54]. The reduction in impact strength after PWHT is attributed to an increase in the density of the segregated precipitates along the inter-dendritic areas. In AW conditions, the weld metal was spotted as the weakest region regarding impact strength, while the P22 base metal was the strongest one. The highest impact strength of the P22 HAZ was attributed to bainitic microstructure; however, the impact strength value was measured lower than the respective base metal. Due to the mixed microstructure of tempered and untempered martensite, P91 HAZ showed an impact strength lower than the P91 base metal. In previous research, a matching P91 filler was used for making the joint of P91/P22 steel and due to the formation of untempered martensite in the weld metal, the welded joint does not meet the specific criteria for impact toughness, i.e., the minimum recommended value of 47 J (ISO 3580:2008) [55]. For the AW joint of P91 and P22 steel with activated TIG and multi-pass TIG process, the impact toughness of the weld metal was reported to be lower than 47 J which was due to the untempered martensite in the weld metal [11,20]. The welded joint with Ni-based filler and austenitic microstructure offered a better impact strength than P91 filler in AW conditions [17]. The acceptable impact strength value for the P22/P91 welded joint was also obtained for the laser welded joint as reported by Sirohi et al. [25]. The weld metal showed a reduction in impact toughness after PWHT for Ni-based filler metal, which might be due to the higher segregation of the secondary phase alloying elements. However, for multi-pass TIG and A-TIG weld joints, a drastic increase in impact strength was reported as a consequence of the PWHT, and it was due to the tempering of the martensite [11,27,56]. From Table 4, it was inferred that the impact strength of P91 and P22 steel HAZ increased drastically after PWHT; however, the impact strength value was measured to be lower than the corresponding base metal. The tempering of the bainitic and martensitic matrix resulted in an increase in impact strength of the P91 and P22 HAZ, respectively. From impact test results for the PWHT joint, it was clear that the weld metal was still poor in terms of impact strength compared with other weldments zones. However, each zone of the weldments fulfills the criteria of minimum impact toughness (47 J) in AW and PWHT conditions.

### 3.5. Residual Stresses

A residual stress measurement was carried out in weld metal using blind hole drilling (BHD) techniques [57]. The magnitude of the longitudinal residual stress was measured at 295 MPa and 135 MPa in the top and root region. A tensile nature of the longitudinal residual stresses was observed in the welded joint’s top and root region. The transverse residual stress magnitude was 250 MPa and 75 MPa in the top and root region, respectively. The behavior of the transverse residual stress was also tensile in nature. The peak magnitude of the longitudinal and transverse residual stresses was measured to be lower than the yield strength of P91 and P22 steel. The magnitude of the residual stresses in the weld metal is mainly governed by shrinkage and volume expansion effects. Generally, volume expansion due to martensitic transformation leads to compressive residual stresses, while the shrinkage effect produces the tensile residual stresses in the weld metal. In the multi-pass welded joint of P91 and P22 steel, the weld metal was solidified as an austenitic microstructure where shrinkage played a major role. That resulted in tensile residual stresses in the weld metal. The martensitic transformation in weld metal mainly occurs for the matching P91 filler, where both shrinkage and volumetric transformation show their significant effect [58]. A similar observation has also been made by Sirohi et al. [59] for the IN617 filler.

## 4. Conclusions

- Inhomogeneity in the microstructure was observed along the weldments for the AW and PWHT joint. The weld metal near the interface solidified as columnar dendrites, while equiaxed dendrites were observed in the centre region of the weld metal. The compositional analysis showed segregation of the Nb and Mo at inert-dendritic areas and along the SGBs while the dendrite core showed the presence of Ni, Cr and Fe as major alloying elements. A wide region of HAZ was observed on both sides of the weld metal and a significant level of heterogeneity in microstructure existed. The interface of between the ERNiCrMo-3 weld metal and base metals was characterized by an unmixed zone (peninsula and island). PWHT had a minute effect on the microstructural behaviour of the austenitic weld metal and interface region while tempering of the martensite and bainite occurred in P91 and P22 HAZ, respectively.
- In AW condition, peak hardness was measured in P91 CGHAZ and it was 440 HV. The hardness of the weld metal was 248 ± 6 HV. At the interface of P91 steel, hardness was measured in a range of 176–259 HV, while at P22 interface, it was 277 HV. After PWHT, both CGHAZ and FGHAZ of P22 and P91 steel showed a drastic reduction in hardness while a region of ICHAZ remained unaffected. The hardness of the weld metal increased from 248 ± 6 HV to 283 ± 8 HV after the PWHT, which might be due to the increase in density of the segregated particles. No carbon migration zones (SZ and HZ) were detected in the AW joint at the interface of P22 steel and ERNiCrMo-3 weld metal. After PWHT, a narrow band of the SZ (130 HV) and HZ (275 HV) formed near the interface P22 steel and ERNiCrMo-3 weld metal and the width of the SZ was measured 310 µm.
- The tensile test specimen showed a failure from the region of P22 base metal for both AW and PWHT joint, which indicates that the weld joint was safe for application in super-critical power plants and that it was also stronger than the base metal. The tensile strength was measured at 615 ± 4 MPa and 493 ± 11 MPa for the AW and PWHT joints, respectively. The reduction in tensile strength after PWHT might be due to over-tempering of the bainite present in P22 steel.
- A variation in impact toughness along the weldments was also measured. However, an acceptable level of impact toughness was measured for the weld metal in both AW and PWHT conditions, i.e., more than the minimum recommended value of 47 J. A difference in impact toughness was also measured for the root and top V-notch impact test. However, weld metal was found to be the weakest region regarding impact strength, while the P22 base metal was the strongest. In the AW joint, the impact strength of the P22 and P91 HAZ was measured at 130 ± 4 J and 75 ± 3 J, respectively, while after PWHT an increase was measured for both the regions.

## Figures and Tables

**Table 1:** Chemical composition of the P91 and P22 base plates and ERNiCrMo-3 filler metal (wt. %).

Element	P91 Base Plate	P22 Base Plate	ERNiCrMo-3 Filler Metal
C	0.08	0.11	0.012
Si	0.25	0.28	0.09
Mn	0.38	0.45	0.37
Cr	8.18	1.95	20.67
Mo	0.90	0.92	9.23
Ni	0.12	0.05	64.89
Nb	0.05	-	3.96
V	0.22	0.09	-
Ti	-	-	0.12
Al	-	-	0.08
Fe	Rest	Rest	0.36

**Table 2:** Welding process parameters.

Position	Welding Current (A)	Arc Voltage (V)
Root pass	125	12–14
Filling pass 1	145	16–18
2	152	20–22
3	152	20–22
4	158	20–22
5	160	22–24
6	160	22–24

**Table 3:** Tensile test results.

Sample		Yield Strength (MPa)		Tensile Strength (MPa)		% Elongation (%e)		Fracture Location	Joint Efficiency (%) [52]
P91 base metal [28]		475 ± 25		715 ± 15		20 ± 2		-	-
P22 base metal		495 ± 5		610 ± 2		35		-	-
AW	Sample 1	420	415 ± 5	619	615 ± 4	27	26.67 ± 0.57	P22 base	86
	Sample 2	415		611		27		P22 base	
	Sample 3	410		615		26		P22 base	
PWHT	Sample 1	384	382 ± 5	505	493 ± 11	27	28.67±1.52	P22 base	69
	Sample 2	376		490		29		P22 base	
	Sample 3	385		484		30		P22 base	

**Table 4:** Impact toughness of the dissimilar welded joint of P91 and P22 steel in AW and PWHT condition.

Impact Toughness	AW	PWHT
Impact toughness (weld metal: top)	65 ± 3 J	58 ± 4 J
Impact toughness (weld metal: root)	74 ± 3 J	69 ± 5 J
Impact toughness (P91 side HAZ)	75 ± 3 J	108 ± 2 J
Impact toughness (P22 side HAZ)	130 ± 4 J	172 ± 5 J

## Data Availability

Not applicable.
